# Essential Oils and Their Constituents: Anticonvulsant Activity 

**DOI:** 10.3390/molecules16032726

**Published:** 2011-03-23

**Authors:** Reinaldo Nóbrega de Almeida, Maria de Fátima Agra, Flávia Negromonte Souto Maior, Damião Pergentino de Sousa

**Affiliations:** 1 Laboratório de Tecnologia Farmacêutica, Federal University of Paraíba, Caixa Postal 5009, CEP 58051-970, João Pessoa, PB, Brazil; E-Mails: agramf@ltf.ufpb.br; (M.-F.A); famaior4@gmail.com (F.-N.S.M.); 2 Department of Physiology, Federal University of Sergipe, CEP 49100-000 São Cristóvão, SE, Brazil; E-Mail: damiao_desousa@yahoo.com.br

**Keywords:** central nervous system, essential oil, antiepileptic drugs, terpenes, medicinal plants

## Abstract

A literature-based survey of plants species and their essential oils with anticonvulsant activity was carried out. As results, 30 species belonging to 13 families and 23 genera were identified for their activities in the experimental models used for anticonvulsant drug screening. Thirty chemical constituents of essential oils with anticonvulsant properties were described. Information on these 30 species is presented together with isolated bioactive compound studies.

## 1. Introduction

According to the WHO [1], about 450 million people in the entire world have suffered mental, neurological, or behavioral problems at some time in their life. Extensive research on plants and their derivatives has taken place in recent years that could provide some new alternative treatments and therapeutic uses for diseases of the central nervous system (CNS).

Epilepsy is the term used for a group of disorders characterized by recurrent spontaneous seizures that apparently result from complex processes involving several neurotransmitter systems such the glutamatergic, cholinergic, and gabaergic system. Actual estimations of the prevalence rate for epilepsy are 1–2% of the world population. Although a considerable number of classic and more modern anticonvulsant drugs are available for the pharmacological treatment of epilepsy patients worldwide, seizures remain refractory in more than 20% of the cases. In addition, all current antiepileptic drugs, which belong to several quite different chemical classes such as hydantoins, deoxybarbiturates, succinimides, benzodiazepines, iminostilbenes and carboxylic acids, have been obtained through chemical synthesis [2].

Several species of aromatic plants are used medicinally because of their volatile oils or chemical components. In particular, some of them possess certain CNS properties, including antiepileptic action and have been traditionally used for a long time in folk medicine. Recent studies on essential oils and their main components have attracted the attention of many scientists and encouraged them to screen any of these natural products to study their chemical and pharmacological aspects that might potentially may lead to lead to the development of new anticonvulsant-like compounds with advantages over current therapeutic drugs [3].

### 1.1. Chemistry of Essential Oils and Main Chemical Constituents

Aromatic plants are at present widely studied for their large therapeutic potential and benefits. These benefits depend largely on essential oils which, in general terms, occur in many herbs. The essential oils of the plant are the essence of their fragrance. They are called essential oils, ethereal oils, or volatile oils because they evaporate quickly when exposed to the air at ordinary temperatures. In general, the essential oils consist of chemical mixtures involving several tens to hundreds of different types of molecules. Only a few have a high percentage of a single component. These chemical constituents are divided into two broad classes: terpenes and phenylpropanoids. However, many volatile oils consist largely of monoterpenes, which are a group of terpenes having ten carbon atoms in the carbon skeleton and, therefore, are composed by two isoprene units [4].

Essential oils are distilled from different parts of the plants including flowers, stem-bark, seeds, leaves, roots, and the whole herbs. They are used to give flavor to foods and drinks and as fragrances in the food and cosmetics industries, where numerous herbal plant and spice ingredients are components in the manufacture of skin creams, lip balms, shampoos, soaps and perfumes. 

## 2. Methodology

The present study was carried out based on the literature review of plants and their essential oils with anticonvulsant activity. All the information about 30 species with anticonvulsant activity is given in Table 1. The list of plants is organized by family and botanical name, parts used and pharmacological activity, as described in the literature. Compounds isolated and references are also provided. The scientific names of the plants were based on W3Tropicos Database (http://mobot.mobot.org/W3T/Search/vast.html), and the abbreviations of author names are according to Brummitt and Powell [5]. 

The plant species presented here were selected based on the effects shown by their essential oils in specific animal models used for evaluation of anticonvulsant activity and/or by complementary studies, aimed at elucidating the mechanism(s) of action of the oils or individual components.

The essential oils or the main constituents were deemed to display anticonvulsant activity when they had shown effects in one or more different seizure model, including the maximal electroshock (MES) model, the pentylenetetrazole seizures model (PTZ), the pilocarpine model and the prolonged PTZ-kindling model.

Some scientific publications that were excluded from this study because it was not possible to access their full text or because their abstracts were in a language different from English, included the following species, citing their psychopharmacological activities: *Apium graveolens* Cham., *Aralia continentalis* Kitag., *Asarum heterotropoides* F. Schmidt*, Asarum himalaicum* Hook. F. & Thomson ex Klotzsch*, Asarum ichangense* C.Y. Cheng & C. S. Yang, *Cumimum cyminum* L, *Eugenia uniflora* L*, Gardenia augusta* (L) Merr., *Gardenia jasminoides* J. Ellis, *Ligusticum sinense* Oliv., *Ocimum basilicum* L*. Oplopanax elatus, Radix bupleuri* and *Salvia sclarea.*

## 3. Results and Discussion

The plant diversity with confirmed activities in the central nervous system is dominated by higher plants, mainly by dicotyledons. In this review 30 species belonging to 13 families and 23 genera have been reported to possess anti-seizure activity. 

The families in decreasing order of predominance of species with activity are Myrtaceae and Lamiaceae with five species each; Apiaceae with four species; Asteraceae and Poaceae with three species each and Araceae and Lauraceae with two species each. Six families, corresponding to 46 % of the total, are represented by only one species. Some of these species have other biological activities and are used for different purposes, like *Egletes viscosa* (L.) Less [6], also mentioned for their antimicrobial activity. 

The predominance of higher plants used for medicinal purposes confirms the results obtained in other ethno-medicinal surveys reported by Agra [7,8]. This has also been documented by authors in different countries around the world such as Brazil [9], Saudi Arabia [10], Bolivia [11] or Italy [12], *inter alia*.

Several aromatic species had been employed since ancient times for their medicinal properties and also as aromatic agents and to give flavor to foods. The pharmacological uses of the plants are mainly attributed to their essential oils having a great variety of pharmacological activities such as prevention and treatment of cancer, against cardiovascular diseases and diabetes. They are also used as sources of gastro-protective, anti-inflammatory, antioxidant, and antibacterial agents. These varied effects are probably due to the high structural diversity of the essential oil constituents. The study of each individual chemical component is critical to understanding its mechanism of pharmacological action and toxicity, including potentially beneficial clinical effects on human health.

However, according to Lahlou [13], the diversity of biological activities presented by the same essential oil has also stimulated discordance between researchers. Many reasons have been proposed for this variability, for example: (a) all the factors that have influence in the chemical composition; (b) the plant’s state of maturation; and (c) the chemotypic difference, among others.

It is accepted that a refined assessment of the chemical composition of tested essential oils/constituents should be performed using GC/MS to perform a quantitative analysis, which would provides additional information of their contents and, consequently, confirmation of their therapeutic effects.

**Figure 1:**
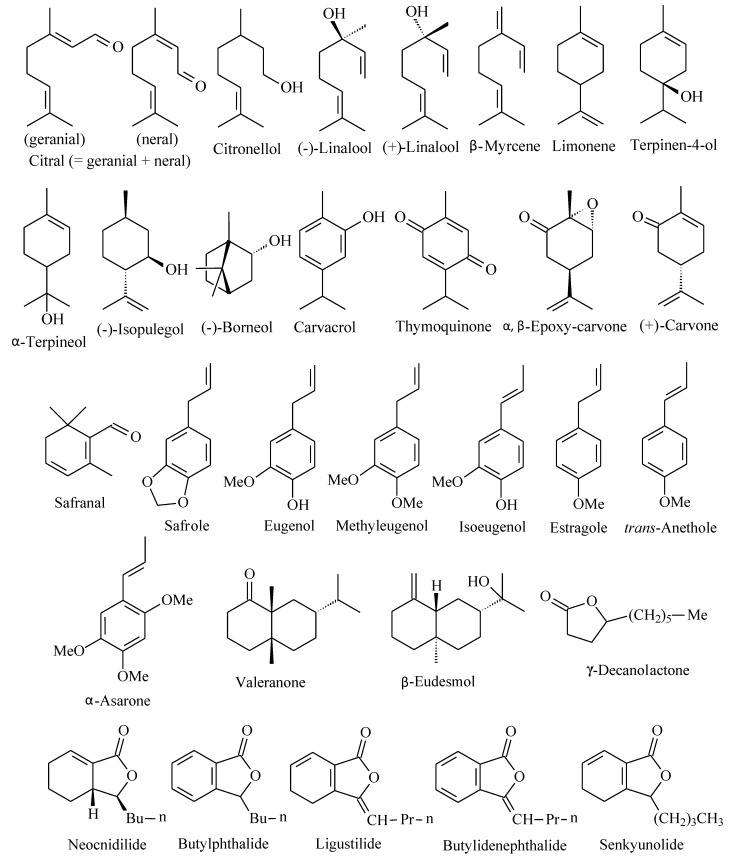
Anticonvulsant chemical constituents of essential oils.

Thirty anticonvulsant chemical constituents of essential oils were mentioned. Most of these compounds are monoterpenes or phenylpropanoids (Figure 1). They are effective in several experimental models of seizure. These constituents must contribute to the anticonvulsant activity of bioactive essential oils, as presented in Table 1. This pharmacological activity may be due to action of a major component or the effect of various bioactive components found in essential oil.

**Table 1:** Species and respective essential oils that showed anticonvulsant activity organized by botanical family, botanical name, part used, activity as described in the literature, compound isolated and references.

FAMILY *Species*	PART USED	ACTIVITY OF ESSENTIAL OILS (as described in the literature)	MAIN COMPOUNDS ISOLATED/REFS.					
**APIACEAE**			
***Cuminum cyminum* Linn. (syn. *Cuminum odorum* Salisb)**	Fruits	The oil showed protection against pentylenetetrazole-induced epileptic activity by increasing the duration, decreasing the amplitude of after hyperpolarization potential (AHP) following the action potential, the peak of action potential, and inhibition of the firing rate.	[14]
***Ferula gumosa* Boiss.**	Fruits	The essential oil protected mice against pentylenetetrazole-induced tonic seizures. The protective dose produced neurotoxicity. Moreover, this dose was too close to the LD_50_ of the essential oil. The anticonvulsant and toxic effects of the essential oil may be related to the compounds pinene and α-thujene respectively.	Pinene andα-thujene [15]
***Heracleum crenatifolium***	Fruits	The essential oil protected mice against maximal electroshock (MES)-induced seizures.	Octyl acetate and octanol [16]
***Pimpinella anisum* L.**	Fruits	Inhibitor of tonic convulsions induced by high doses of pentylenetetrazole and electroshock trans-corneal.	[17]
**ARACEAE**			
***Acorus calamus* L.**	Not available	Anticonvulsant action against experimental electroshock. Neither essential oil nor diphenylhydantoin were effective in modifying convulsions produced by metrazole.	[18]
***Acorus gramineus* Aiton**	Rhizomes	Inhibited the specific bindings of a use-dependent NMDA receptor-ion. Neuroprotective effects on cultured cortical neurons through the blockade of NMDA receptor activity.	[19]
	Rhizomes	Anticonvulsant effects, both *Acorus gramineus* and α-asarone can enhance the reactivity and convulsive threshold of immature rats to electric stimulation.	α-asarone [20]
	Rhizomes	Pre-inhalation of the oil markedly delayed the appearance of pentylene-tetrazole-induced convulsion. Furthermore, inhalation impressively inhibited the activity of gamma-aminobutyric acid (GABA) *trans*-aminase, a degrading enzyme for GABA as the inhalation period was lengthened. The GABA level was significantly increased and glutamate content was significantly decreased in mouse brain by pre-inhalation of the essential oil.	[21]
***Acorus tatarinowii* Schott.**	Rhizomes	The volatile oil (1.25 g/kg) decreased the convulsive rate significantly in the maximal electroshock model. But failed to prevent seizures in the dose range tested, although prolonged seizure latency and decreased mortality were found at a dose of 1.25 g/kg. The oil can prevent convulsions as well as convulsion-related GABAergic neuron damage in the brain in the prolonged pentylenetetrazol kindling model.	[22]
**ASTERACEAE**			
***Artemisia annua* L.**	Fresh leaves	The essential oil (AEO) obtained by hidrodestilation increased the latency time to convulsions induced by picrotoxin and pilocarpine but prevented the onset of pentylenotetrazol and strychnine induced seizures.	[23]
***Artemisia dracunculus* L.**	Aerial parts	The oil exerted dose and time-dependent antiseizure activity reported in both maximal electroshock and pentylenetetrazole models.	*trans*-Anethole, α-*trans*-ocimene, limonene, α-pinene, cymene, eugenol, β-pinene, α-terpinolene, bornyl acetate, and bicyclogermacrene [24]
***Egletes viscosa* (L.) Less.**	Inflorescences	Activity against convulsion induced by PTZ in mice.	[6]
**EUPHORBIACEAE**			
***Croton zehntneri*** **Pax & K. Hoffm.**	Not available	Elevation of the threshold for starting a minimal convulsions induced by pentylenetetrazol.	[25]
**FABACEAE-MIMOSOIDEAE**			
***Tetrapleura tetraptera*** **(Schumach. & Thonn.) Taub.**	Fruits	Inhibitor of convulsions induced by PTZ and electroshocking in mice of both sex.	[26]
	Fresh fruits	The fresh oil given intraperitoneally offers some protection against leptazol-induced convulsions. A dose of 0.4 ml of the oil per mouse protected 78% of the animals when administered 30 min prior to leptazol	[27]
**LAMIACEAE**			
***Aeollanthus suaveolens* Mart. ex Spreng.**	Not available	Inhibitor effect of convulsions induced by pentylenetetrazol and maximal electroshock in mice	Linalool[28]
	Leaves	The anticonvulsant properties of γ-decanolactone was observed in mice. The neurochemical essay with linalool in cortical membranes of rats showed a dose-dependent, not competitive of *binding* of [^3^H] MK 801 – an antagonist of receptor NMDA. Also observed the effects of linalool on glutamatergic system in the rat cerebral cortex and that linalool modifies the nicotinic receptor – ion channel kinetics at the mouse neuromuscular junction.	γ-Decanolactone and linalool [29,30,31,32]
	Not available	The oil has an inhibitory effect of linalool on glutamate binding in rat cortex was observed.	Linalool [30]
	Not available	Sedative properties on mice and has dose-dependent marked effects on the central nervous system, including hypnotic, anticonvulsant and hypothermic activity	γ-Decanolactone [29]
	Not available	An inhibitory effect of linalool on the acetylcholine (ACh) release and on the channel open time in the mouse neuromuscular junction.	Linalool [31]
***Hyptis suaveolens* (L.) Poit.**	Leaves	Anti-convulsive in tests induced by pentylenetetrazol and electroshock in rats of both sex	[26]
***Lavandula stoechas* L.**	Not available	Used as inhalator showed anti-convulsive activity similar to the above. Moreover, was verified a higher level of latency and a reduction of level of the severity of convulsions. Complementary test suggest this activity maybe is related with the blocking of canals of calcium. The inhaling lavender oil vapor blocked pentylene-tetrazole- and nicotine-induced convulsion and electroshock convulsion in mice.	[33]
***Ocimum gratissimum* L.**	Not available	Essential oil obtained in Spring was able to protect animals against tonic seizures induced by electroshock (MES, 50 mA, 0.11 s).	Eugenol [34]
***Ocimum basilicum***	Aerial part	When tested in mice, the essential oil, higher doses, produced significantly increased in a dose-dependent manner the latency of convulsion and percent of animals exhibiting clonic seizures. Likewise, it reduced lethality in response to different convulsive stimulus used in this study.	[35]
***Ocimum basilicum***	Leaves	Essential oil increased the latency for development of convulsions in pentylenetetrazol and PIC tests. For pentylenetetrazol, the effects of EO were reversed by flumazenil. EO did not interfered with the convulsions induced by strychnine.	1.8-Cineole, linalool, and geraniol were the main components, comprising 92.9% of the oil. [36]
	Not available	Essential oil blocked the clonic seizures induced by pentylenetetrazole, picrotoxin and strychnine	[37]
**LAURACEAE**			
***Laurus nobilis* L.**	Leaves	Anticonvulsant activity was observed against pentylenetetrazole- and maximal electroshock-induced seizures. At anticonvulsant doses, the essential oil produced sedation and motor impairment.	Methyleugenol, eugenol and pinene [38]
***Myristica fragrans***	Seed	Nutmeg oil was found to possess significant anticonvulsant activity against electroshock-induced hind limb tonic extension. It exhibited dose dependent anticonvulsant activity against pentylenetetrazole-induced tonic seizures. It delayed the onset of hind limb tonic extensor jerks induced by strychnine. Also it was anticonvulsant at lower doses, whereas weak proconvulsant at a higher dose against pentylenetetrazole and bicuculline induced clonic seizures.	[39]
**MYRTACEAE**			
***Eucalyptus citriodora* Hook**	Leaves	Not available	Citronellal, citronellol, and citronellyl acetate [40]
***Eucalyptus urophylla***	Leaves	The oil increased the number of mice protected against pentylenetetrazole-induced death	[41]
***Eucalyptus camaldulensis* var. camaldulensis Dehn.**	Leaves	Not available	*p*-Cymene, spathulenol, cryptone, thymol, and linalool [42]
***Syzygium aromaticum* (L.) Merr. & L.M. Perry** *=Eugenia caryophyllata* Thunb.	Flowers	Inhibition of tonic convulsions induced by electroshock in rats	[43]
***Psidium persoonii* McVaugh** ***=*** *Psidium guianense* **Pers.**	Leaves	The doses of 100, 200, and 400 mg/kg, by via oral reduced as dose-dependent the severity of convulsions induced by pentylenetetrazole. Moreover, induced the depressor effect of spontaneous movement.	[44,45,46]
**POACEAE**			
***Cymbopogon winterianus*** **Jowitt**	Leaves	Anticonvulsant activity was observed in pentylenetetrazole, pilocarpine, and strychnine tests. Anticonvulsant effect was blocked by flumazenil in pentylenetetrazole model.	The EO showed presence of geraniol, citronellal, and citronellol as the main compounds. [47], [48]
***Cymbopogon citratus*** **(DC)Stapf**	Leaves	Anticonvulsant activity was observed in pentylenetetrazole, pilocarpine, strychnine, and maximal electroshock tests.	[47], [49]
***Cymbopogon proximus***	Plant	Administration of the oil to mice before induction of convulsions with electroshock, resulted in complete protection. There was partial protection in pentylenetetrazole, picrotoxin and strychnine tests.	Piperitone, elemol, and eudesmol. [50]
**RANUNCULACEAE**			
***Nigella sativa*** **L.**	Not available	The oil was the most effective in preventing pentylenetetrazole-induced seizures relative to valproate; also showed significantly decreased oxidative injury in the mouse brain tissue in comparison with the pentylenetetrazole-kindling group	Thymoquinone [51]
**RUTACEAE**			
***Citrus aurantium* L.**	Peel from fruits	EO from peel increased the latency period of tonic seizures in pentylenetetrazol and maximal electroshock models. Effect was not dose-dependent	[52]
**VALERIANACEAE**			
***Nardostachys jatamansi*** **(D. Don) DC.**	Not available	A ketonic principle, jatamansone, isolated from oil of *N. jatamansi*, is more effective than quinidine and essential oil of jatamansi in suppression of ectopic ventricular activity in unanesthetized dogs produced by 2-stage coronary ligation, equal to quinidine in combating auricular flutter induced by injury stimulation, and more effective than Na diphenyl-hydantoin and essential oil of jatamansi in maximal electroshock seizures.	Jatamansone [53]
**VERBENACEAE**			
***Lippia alba* (Mill.) N.E. Brown.**	Not available	The anticonvulsive effects of the essential oils (EOs) from three chemotypes of *Lippia alba* was observed. The animals were treated with citral (100 mg/kg, i.p.), α-myrcene or limonene (200 mg/kg, i.p.), EOs chem. constituents, presented significant increases in the latency of convulsion and percentage of survival. The association of EOs with diazepam significantly potentiated their effects, suggesting a similar mechanism of action.	Citral β-myrceneLimonene [54]

Considering the thirty species selected in this study, anticonvulsant effect was observed in twenty four of them using the PTZ model. The prevention of seizures induced by PTZ in laboratory animals is the principal protocol used to characterize a potential anticonvulsant drug. The PTZ test represents a valid model for human generalized myoclonic seizures and also by the absence of seizures. On other hand, seventeen species showed anticonvulsant effect by blocking tonic convulsions induced by MES. This test is considered to be a predictor of anti-epileptic drug activity against generalized tonic-clonic seizures.

The more frequent studies involved substances tested via an intraperitoneal route. However, essential oils of *Acorus gramineus* Aiton and *Lavandula stoechas* L. only showed anticonvulsant activity when administered by inhalation. This pharmacological result is very interesting as it represents a non invasive route. In a recent review, Edris [55] also presented some comments about the effects of substances administered by inhalation. Additionally, have been suggestions that the inhibitory effect on the central nervous system occurs via gamma-aminobutyric acid (GABA)-ergic neuromodulation system.

The anticonvulsant effect of the main chemical constituents of essential oils (Figure 1) have also been studied. The natural abundance of these compounds allowed systematic studies on their pharmacological properties. Most studies report antiseizure activity of these compounds in animal models of convulsion. The results showed that common essential oil constituents such as terpinen-4-ol [56,57], citral, β-myrcene, limonene [53,58], safranal [59,60], linalool [27,61], γ-decanolactone [28,62], α-terpineol [63], (-)-isopulegol [64], citronellol [65], thymoquinone [66], α,β-epoxycarvone [67], (*S*)-(+)-carvone [68], eugenol, methyleugenol, isoeugenol, estragole, safrole [69], *trans*-anethole [70], (-)-borneol and carvacrol [58] are all bioactive in experimental models. Interesting, (*R*)-(-)-carvone had no anticonvulsant effect. The study showed that the chiral center at carbon 4 in the carvone molecule is important in the interaction with the receptor. The molecule with isopropenyl group in *S* configuration at carbon 4 is clearly capable of reducing the convulsive effect of PTZ and PIC in terms of onset time. A comparative analysis of the constituents cannot however be done due to the different experimental conditions and variability of methods and routes of administration used in the evaluation of these compounds.

Anticonvulsant activity was also identified in some sesquiterpenes such as valeranone [52] and β-eudesmol [71]. Others compounds found in essential oils also showed this effect. For example, some phthalides such as butylidenephthalide, ligustilide, butylphthalide, neocnidilide, and senkyunolide [72]. α-Asarone, a phenylpropanoid, also presented effective anticonvulsant activity [19].

The present study reports a limited number of plant species based on a set of papers which the molecular mechanisms of anticonvulsant action of the volatile oils/constituents was possible to investigate. Of these, two species are prominent, *Acorus gramineus* Aiton and *Aeollanthus suaveolens* Mart. ex Spreng, belonging to the Araceae and Lamiaceae families, respectively. 

The activity of *Acorus gramineus* was suggested by the essential oil having anticonvulsant effect through the blockade of NMDA receptor. On other hand, the psychopharmacological effect of *Aeollanthus suaveolens* was attributed to the monoterpene compound linalool, reported to be a major component of essential oils in several aromatic plants, including this species. Recent findings provide evidence that the anticonvulsant effects of monoterpenes could be modulated by Ach mechanisms. Moreover, other studies suggest a direct interaction with the NMDA receptor complex [31].

## 4. Conclusions

In conclusion, the thirty aromatic species, listed in the present paper and represented by their essential oils/chemical constituents, appear to be promissory as sources of anticonvulsant agents. Thirty anticonvulsant chemical constituents of essential oils also were related. Nevertheless, the data show that most chemical classes of compounds found in essential oils are effective in convulsion models. They must act by different mechanisms of action that merit further investigation. The future outlook for the development of new antiepileptic drugs derived from essential oils is therefore positive.
